# Antioxidants against contrast media induced nephrotoxicity

**DOI:** 10.12861/jrip.2014.18

**Published:** 2014-04-10

**Authors:** Majid Tavafi

**Affiliations:** Department of anatomy, Faculty of Medicine, Lorestan University of Medical sciences, Khoram Abad, Iran

**Keywords:** Contrast media, Nephrotoxicity, Antioxidant

**Implication for health policy/practice/research/medical education:**

Although intravascular injection of contrast media is very important in diagnostic radiology but may lead to acute renal failure and hospitalization. It seems that, pathogenesis of contrast media nephrotoxicity acts through renal vasoconstriction, medullary ischemia, tubular cell death and production of reactive oxygen species. Pretreatment with antioxidants attenuated renal side effects of contrast media in patients and laboratory animals.

Radiological procedures using injection of contrast media have been employed increasingly for diagnostic purposes (1). Nevertheless, side effect of contrast media injection known as contrast media nephrotoxicity is still a major complication and it has became the third leading cause of acute renal failure, needing hospitalization (2). Despite enormous researches, the nephrotoxicity of radiographic contrast agents remains a serious clinical problem. Proteinuria and an increase in serum creatinine and urea are the clinical features of contrast media nephrotoxicity. The structural side effects of contrast media include: vacuolization of proximal tubules cells, DNA fragmentation (apoptosis activation) and necrosis of the thick ascending limbs cells of Henle’s loops (3), interstitial inflammation and enzymuria (4). Besides, high-osmolar contrast media increased progression of glomerulosclerosis in old, spontaneously hypertensive male rats (5). The pathogenesis of contrast media nephrotoxicity is not completely clear. Proximal and distal tubular injury occurs at the moment of contact with contrast medium and is thought to be due to intrarenal vasoconstriction, medullary hypoxia (ischemia), and direct tubular cell death. Contrast agents may trigger the release of endothelin and adenosine from endothelial cells, increasing vasoconstriction, decreasing the release of prostaglandins and preventing vasodilatation, thus decreasing oxygen in the outer medulla (3,6,7). Hypoxia and decreased nutrient delivery to tubular epithelial cells, resulting in increases in reactive oxygen species, causing breakdown of the epithelial cytostructure and death of the cell (6,8). Based on pathogenesis prevention strategies, should focus on counteracting vasoconstriction, enhancing blood flow and oxygen of renal medulla, and also protection against oxygen free radicals via antioxidants usage (9). Different antioxidants were used against contrast media nephrotoxicity in human and rodent (10,11). Recently, Nasri *et al*. used green tea (*Camellia sinensis*) extract in combat with contrast media nephrotoxicity in rats for the first time (12). They showed that green tea extract ameliorated renal functional tests such as serum creatinine and blood urea nitrogen in rats that received contrast media. Their results may be due to phenolic antioxidants and anti-inflammatory compounds that exist in green tea extract. Hence, using green tea extract and other plant antioxidants in human and researches against nephrotoxic agents such as contrast media, cisplatin or gentamicin sulphate is suggested.

## Acknowledgements

The author wants to thank from Rezvan Bagheri.

## Author’s contribution

MT is the single author of the manuscript.

## Conflict of interests

The author declared no competing interests.

## Ethical considerations

Ethical issues (including plagiarism, data fabrication, double publication) have been completely observed by the author.

## Funding/Support

None declared.
